# T_1ρ_-mapping for assessing knee joint cartilage in children with juvenile idiopathic arthritis — feasibility and repeatability

**DOI:** 10.1007/s00247-019-04557-4

**Published:** 2019-11-09

**Authors:** Anouk M. Barendregt, Valentina Mazzoli, J. Merlijn van den Berg, Taco W. Kuijpers, Mario Maas, Aart J. Nederveen, Robert Hemke

**Affiliations:** 1 Department of Radiology and Nuclear Medicine, Amsterdam University Medical Centers, location AMC, Meibergdreef 9, Room G1-226, 1105 AZ Amsterdam, The Netherlands; 2 Amsterdam University Medical Centers, Department of Pediatric Hematology, Immunology, Rheumatology and Infectious Disease, Emma Children’s Hospital, Amsterdam, The Netherlands; 3 Department of Radiology, Stanford University, Stanford, CA USA

**Keywords:** Adolescents, Arthritis, Cartilage, Children, Gadolinium, Juvenile idiopathic arthritis, Magnetic resonance imaging, Quantification, T_1ρ_

## Abstract

**Background:**

Ongoing arthritis in children with juvenile idiopathic arthritis (JIA) can result in cartilage damage.

**Objective:**

To study the feasibility and repeatability of T_1ρ_ for assessing knee cartilage in JIA and also to describe T_1ρ_ values and study correlation between T_1ρ_ and conventional MRI scores for disease activity.

**Materials and methods:**

Thirteen children with JIA or suspected JIA underwent 3-tesla (T) knee MRI that included conventional sequences and a T_1ρ_ sequence. Segmentation of knee cartilage was carried out on T_1ρ_ images. We used intraclass correlation coefficient to study the repeatability of segmentation in a subset of five children. We used the juvenile arthritis MRI scoring system to discriminate inflamed from non-inflamed knees. The Mann-Whitney *U* and Spearman correlation compared T_1ρ_ between children with and without arthritis on MRI and correlated T_1ρ_ with the juvenile arthritis MRI score.

**Results:**

All children successfully completed the MRI examination. No images were excluded because of poor quality. Repeatability of T_1ρ_ measurement had an intraclass correlation coefficient (ICC) of 0.99 (*P*<0.001). We observed no structural cartilage damage and found no differences in T_1ρ_ between children with (*n*=7) and without (*n*=6) inflamed knees (37.8 ms vs. 31.7 ms, *P*=0.20). However, we observed a moderate correlation between T_1ρ_ values and the juvenile arthritis MRI synovitis score (r=0.59, *P*=0.04).

**Conclusion:**

This pilot study suggests that T_1ρ_ is a feasible and repeatable quantitative imaging technique in children. T_1ρ_ values were associated with the juvenile arthritis MRI synovitis score.

## Introduction

Juvenile idiopathic arthritis is the most common rheumatic disease in childhood [1]. Cartilage can be damaged by the autoimmune-mediated inflammation that originates in the synovial membrane. Ongoing inflammation can subsequently extend to the cartilage and result in degradation of cartilage and bone matrix. Osteochondral damage is presumably irreversible and associated with disability and decreased quality of life [2, 3]. Previous research also indicates that damaged cartilage matrix facilitates binding of synovial inflammatory cells [4]. Thus, even when inflammation subsides, a damaged matrix leaves the child at increased risk for more cartilage degradation whenever a flare occurs. Damage to the cartilage matrix is characterized by loss of proteoglycan and collagen, as demonstrated in studies focusing on rheumatoid arthritis [5–9]. This microstructural damage is important to recognize because intensification of anti-rheumatic treatment might prevent irreversible cartilage damage. Current imaging techniques, such as radiography, US imaging and conventional MRI, can detect structural bone damage and synovial inflammation but cannot detect microstructural damage to cartilage [10].

T_1ρ_ is an MRI parameter that is hypothesized to quantify proteoglycan loss of the cartilage matrix in vitro [11] and in vivo [12]. T_1ρ_ uses a weak radiofrequency pulse to lock protons in phase, which slows transverse relaxation and reduces the effect of dipolar interactions. As a result, the measured relaxation of protons can be attributed to time-constant T_1ρ_. Earlier studies in osteoarthritis and rheumatoid arthritis [12–16] showed that T_1ρ_ values increase as a consequence of decreased proteoglycan content. Cartilage integrity was studied once in pediatric patients using T_1ρ_: knee cartilage of 10 healthy children was assessed and a mean T_1ρ_ value of 76.6 ms was reported [17]. Cartilage in children is distinct from articular cartilage in adults. Postnatally, chondrocytes are small and there is a scant matrix without a distinct zonal orientation [18]. Animal studies demonstrated that proteoglycan content is highest postnatally, with a gradual decrease in proteoglycans during aging, while collagen content increases and collagen fibers show increasing isotropy, resulting in development of a zonal organization of the cartilage [19–21].

The primary aim of this pilot study was to evaluate feasibility (motion artefacts and patient comfort) of T_1ρ_ knee cartilage imaging in children with juvenile idiopathic arthritis; secondarily we studied repeatability of T_1ρ_ values derived by manual cartilage segmentation. Moreover, we explored T_1ρ_ values in juvenile idiopathic arthritis by comparing T_1ρ_ values with conventional MRI scores using the juvenile arthritis MRI scoring system [22].

## Materials and methods

### Patients

In this pilot study, we included all consecutive pediatric patients who underwent MRI of the knee between April 2016 and August 2016 in the Academic Medical Center/Univerisity of Amsterdam, Amsterdam, The Netherlands. Patients visited one of the outpatient clinics of three tertiary pediatric rheumatology centers the Academic Medical Center/Univerisity of Amsterdam, OLVG hospital and Reade, all in Amsterdam, the Netherlands. Patients were clinically assessed by one of our pediatric rheumatologists J.M. van den Berg, D. Schonenberg-Meinema, A. Nassar-Sheikh Rashid and K.M. Dolman. Clinical characteristics consisted of age, height, weight, body mass index (adjusted for age and gender using body mass index percentiles and body mass index z-scores [23], calculated using the pediatric z-score calculator from the Children’s Hospital of Philadelphia [24]); a global assessment of disease activity measured on a 0–100 visual analogue scale by the physician; and an 84-joint count evaluating the number of actively inflamed joints. Laboratory tests included the erythrocyte sedimentation rate, which is the most widely used laboratory marker for juvenile idiopathic arthritis-related inflammation [25], and C-reactive protein.

Inclusion criteria for this pilot study were (1) clinically active arthritis (defined as joint swelling or limitation of motion, with pain or tenderness to palpation) involving at least one knee in children with juvenile idiopathic arthritis or suspected new-onset juvenile idiopathic arthritis; or (2) follow-up of children with juvenile idiopathic arthritis with clinically inactive disease who had a history of clinically evident arthritis in at least one knee.

Exclusion criteria were intra-articular corticosteroid injection within the last 6 months, the need for anesthesia during MRI examination and general contraindications for MRI. Written informed consent was obtained from all parents and, if the patient was 12 years or older, written informed consent was also obtained from the patient, as prescribed by local ethics regulations.

### Magnetic resonance imaging protocol

Knee MR images were obtained using a 3.0-T magnet (Ingenia; Philips Medical Systems, Best, the Netherlands) and comprised all sequences of our standard juvenile idiopathic arthritis MRI protocol and in addition a sagittal T_1ρ_, which was acquired before administration of contrast agent (Table 1). To measure T_1ρ_ we used a B0/B1-compensated T_1ρ_-prepared 3-D gradient echo sequence. Each spin lock pulse consisted of two continuous radiofrequency pulses with opposite phase to compensate for B1 variations. A 180° refocusing pulse was placed between the two continuous radiofrequency pulses to compensate for B0 inhomogeneities [26, 27]. We repeated the sequence with five spin lock times (5 ms, 10 ms, 20 ms, 40 ms and 70 ms), spin lock frequency of 400 Hz and imaging acceleration sensitivity encoding factor 2. The acquisition time for a single spin lock time was 1 min 51 s, resulting in a total acquisition time of 9 min 15 s for the complete T_1ρ_ scan. Scans were made in non-loading conditions with children placed in supine position with the knee in the center of the scanner bore. A dedicated 16-channel transmit/receive knee coil was used. To standardize the influence of potential biomechanical load [28, 29], we kept loading differences prior to the MRI examination to a minimum by preparing all patients equally: 1 h before MRI, children were invited in the hospital for intravenous cannula placement, and subsequently they were taken to the radiology department by one of our researchers using the elevator. All children walked a similar distance of maximum 500 m and did not participate in other knee-loading activities in the hour prior to the MRI.

**Table 1:** Three-tesla magnetic resonance imaging sequences and parameters of our standard juvenile idiopathic arthritis MRI protocol and of the additional sagittal T_1ρ_

	Gadolinium	Repetition time (ms)	Echo time (ms)	Field of view	Voxel size (mm)	Slice thickness (mm)	Number of signal averages	Flip angle
Sagittal turbo spin-echo proton density	–	2,000	40	160×160	0.25×0.25	3.0	1	90°
Sagittal turbo spin-echo 3-D T2 spectral attenuated inversion recovery	–	1,500	250	144×144	0.5×0.5	0.7	2	90°
Sagittal 3-D turbo spin-echo T1 spectral attenuated inversion recovery	–	400	73	168×168	0.5×0.5	0.8	2	90°
Sagittal *T*_*1*ρ_	–	3.6	2.09	150×150	0.6×0.6	2.0	1	15°
Sagittal 3-D turbo spin-echo T1 spectral attenuated inversion recovery	+	400	73	168×168	0.5×0.5	0.8	2	90°

### Image analysis

The conventional MR image sets were scored by an experienced reader R. Hemke (RH), with 7 years’ experience in musculoskeletal radiology) who was blinded to the clinical history of the patients. The reader scored the presence and extent of disease activity and osteochondral damage using the validated juvenile arthritis MRI scoring system, which has been described in detail [22].

### T_1ρ_ post-processing

After image acquisition the T_1ρ_ images acquired at longer spin lock times were registered to the T_1ρ_ images of the corresponding shortest spin lock time (5 ms) using an affine registration pipeline implemented in elastix [30]. This was done to correct for small subject motion occurring between the different scans. The T_1ρ_ relaxation maps were obtained by fitting the signal intensities of the registered images to a three-parameters exponential decay model in a pixel-by-pixel fashion.

### Cartilage segmentation

The total articular cartilage of the knee was manually segmented into eight regions of interest by an experienced reader (RH, with 7 years’ experience in musculoskeletal radiology) on the T_1ρ_ images acquired with spin lock time of 5 ms using ITK-SNAP [31]. Segmentation was performed using the conventional axial, sagittal and coronal images to ensure accurate segmentation of the cartilage. The regions of interest reflected eight anatomical cartilage regions following the juvenile arthritis MRI scoring system [22]. T_1ρ_ values in milliseconds were extracted per region of interest and then combined, resulting in mean T_1ρ_ values of the total articular cartilage per patient. Moreover, the regions of interest were sub-grouped into weight-bearing cartilage (medial and lateral tibial plateau, medial and lateral weight-bearing femur) or non-weight-bearing cartilage (medial and lateral patella, medial and lateral anterior part of condyle) resulting in mean T_1ρ_ values for the weight-bearing cartilage and non-weight-bearing cartilage.

### Reliability of repeated cartilage segmentation

To analyze the repeatability of the delineation of the articular knee cartilage on T_1ρ_, the same reader [blinded] performed the delineation process a second time in five randomly selected patients, 4 months after the first delineations.

### Statistical analysis

All analyses were carried out using SPSS Statistics version 24.0 (IBM Corp., Armonk, NY). We studied normality of the data and whether the data had a non-Gaussian distribution; medians and interquartile range were reported and non-parametric tests were used for analysis. A *P*-value <0.05 was considered to indicate a statistically significant difference. First, descriptive characteristics of the patients were reported. Second, we studied the intra-reader reliability in five children who were segmented twice by the same reader using the intraclass correlation coefficient in a two-way mixed model with single measures. To further gain insight into T_1ρ_ differences between the first and second segmentation, we created a Bland–Altman plot and calculated and the coefficient of repeatability (1.96 × standard deviation of the differences) [32]. Next, we studied the difference between total mean T_1ρ_ values and individual region-of-interest T_1ρ_ values in MRI-active disease (defined as children with a juvenile arthritis MRI score ≥1) as compared to MRI-inactive disease (defined as children with a juvenile arthritis MRI score of 0) and we studied the difference in T_1ρ_ values between weight-bearing and non-weight-bearing cartilage, all using Mann–Whitney *U* test. Subsequently, we studied correlation between the T_1ρ_ value and disease activity parameters (juvenile arthritis MRI score, erythrocyte sedimentation rate, number of actively inflamed joints) with a Spearman ρ test. Last, we studied correlation between T_1ρ_ values and age and body mass index of the patients with a Spearman ρ test.

## Results

### Patients

From April 2016 to August 2016, we included 13 children (9/13 or 69% girls; median age 13.7 years, interquartile range [IQR] 11.6–15.9 years). Demographic and clinical characteristics can be found in Table 2. In five children with suspected new-onset juvenile idiopathic arthritis at the date of MRI, the diagnosis juvenile idiopathic arthritis was not confirmed. They were diagnosed with reactive arthritis, panuveitis with arthralgia, hypermobility and pain syndrome (*n*=2), respectively.

**Table 2:** Patient characteristics and disease activity markers of the study cohort

	MRI-inactive patients (juvenile arthritis MRI score 0, *n*=6)	MRI-active patients (juvenile arthritis MRI score ≥1, *n*=7)
Age	13.8 (13.6–15.6)	12.2 (10.2–16.8)
Body mass index percentile	23 (12–43)	59 (46–93)
Body mass index z-score	0.215 (0.05–1.46)	−0.74 (−1.18 to −0.17)
Number of females (%)	5 (83%)	4 (57%)
Active joints at physical examination	1 (0.0–2.3)	1 (0.0–4.0)
Joints with limitation of motion at physical examination	1 (0.8–1.0)	1 (0.0–2.0)
Erythrocyte sedimentation rate in mm/h	5 (2.0–7.3)	8 (6.0–22.0)
C-reactive protein in mg/L	0.9 (0.2–2.3)	2.1 (0.0–4.6)
Visual analogue scale physician	4 (0.0–8.8)	15 (8.0–20.0)
Total juvenile arthritis MRI score	0 (0–0)	3 (1–10)

### Feasibility

**Fig. 1:**
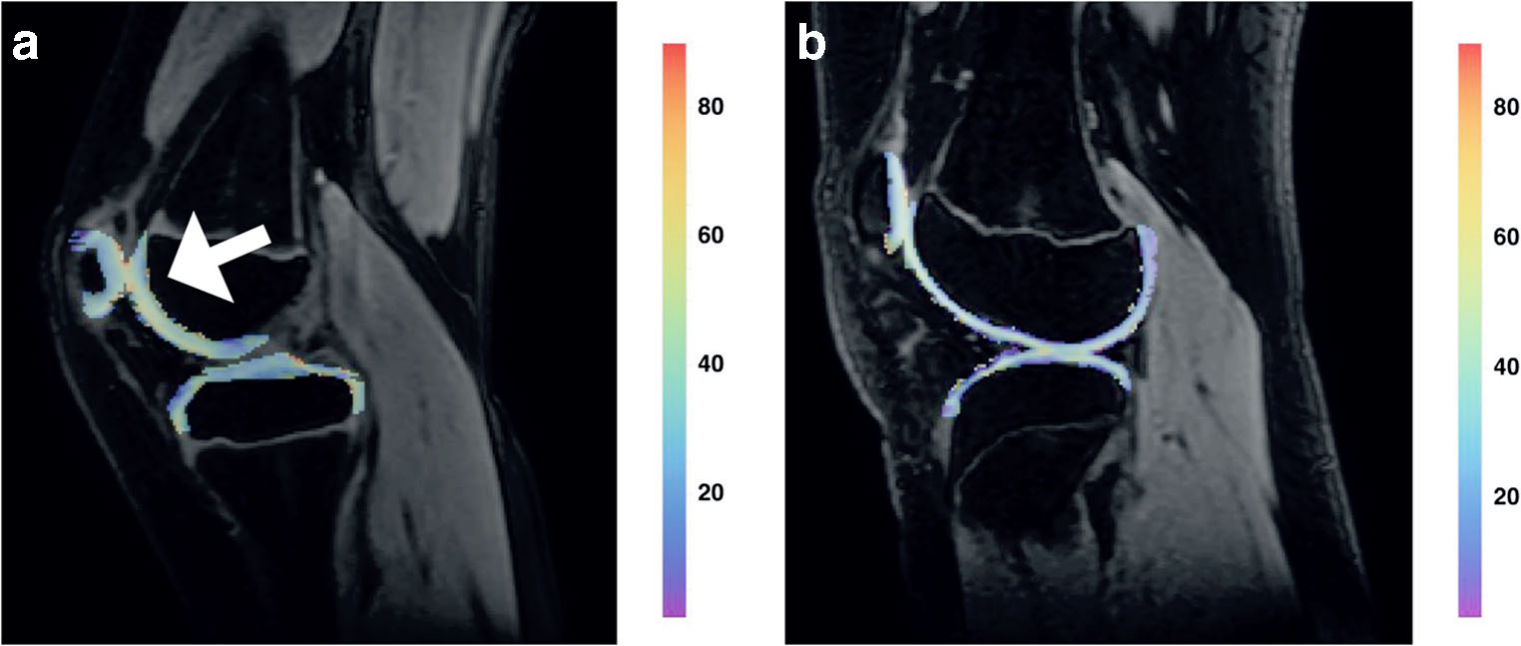
Two T_1ρ_ MR imaging examples in children from this study. **a, b** T_1ρ_ overlay image (**a**) of a 10-year-old boy with oligo-articular juvenile idiopathic arthritis who had a swollen and warm right knee with slight limitation of motion on examination. On the image, higher T_1ρ_ values are observed as compared to the child depicted in (b). Especially the cartilage in the patellofemoral joint demonstrates high T_1ρ_ values (*arrow*). In this boy, synovial inflammation was observed on MR imaging in the patellofemoral synovium and around the cruciate ligaments. T_1ρ_ overlay image (**b**) of a 13-year-old girl who presented with knee pain. She was suspected of having juvenile idiopathic arthritis. On examination, minor swelling of the knee was found. The conventional MRI showed no synovial inflammation in the knee. T_1ρ_ values are markedly lower as compared to (a). Note the higher relative contribution of purple and blue in the cartilage overlay. After clinical, laboratory and imaging evaluation she was diagnosed with a functional pain syndrome

All 13 children underwent T_1ρ_ MRI scanning without discomfort and the total MR protocol was acquired without any problems. No movement artifacts were observed and all acquired T_1ρ_ images were of sufficient quality for post-processing. Figure 1 depicts conventional and T_1ρ_ MR imaging examples.

### Repeatability of segmentation-derived T_1ρ_ values

**Fig. 2:**
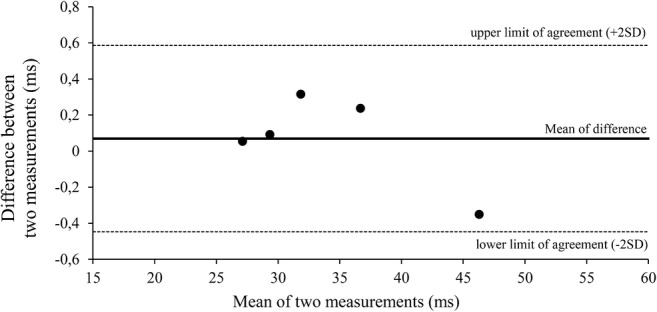
Bland-Altman plot shows the difference between the first and second T_1ρ_ measurements as measured in a sample of five randomly selected children

To study repeatability of the T_1ρ_ values that were derived from manual knee cartilage segmentations, the reader repeated the segmentation in five randomly selected children 4 months after the first segmentations. The intraclass correlation coefficient of the T_1ρ_ values was 0.99 (95% confidence interval [CI] 0.99–1.00, *P*-value <0.001). For further analysis, we created a Bland–Altman plot (Fig. 2), which showed small differences between measurements. Coefficient of repeatability was 0.51 ms, which is a difference of <2% from the mean T_1ρ_ value.

### T_1ρ_ values in magnetic resonance imaging (MRI)-active versus MRI-inactive disease

**Fig. 3:**
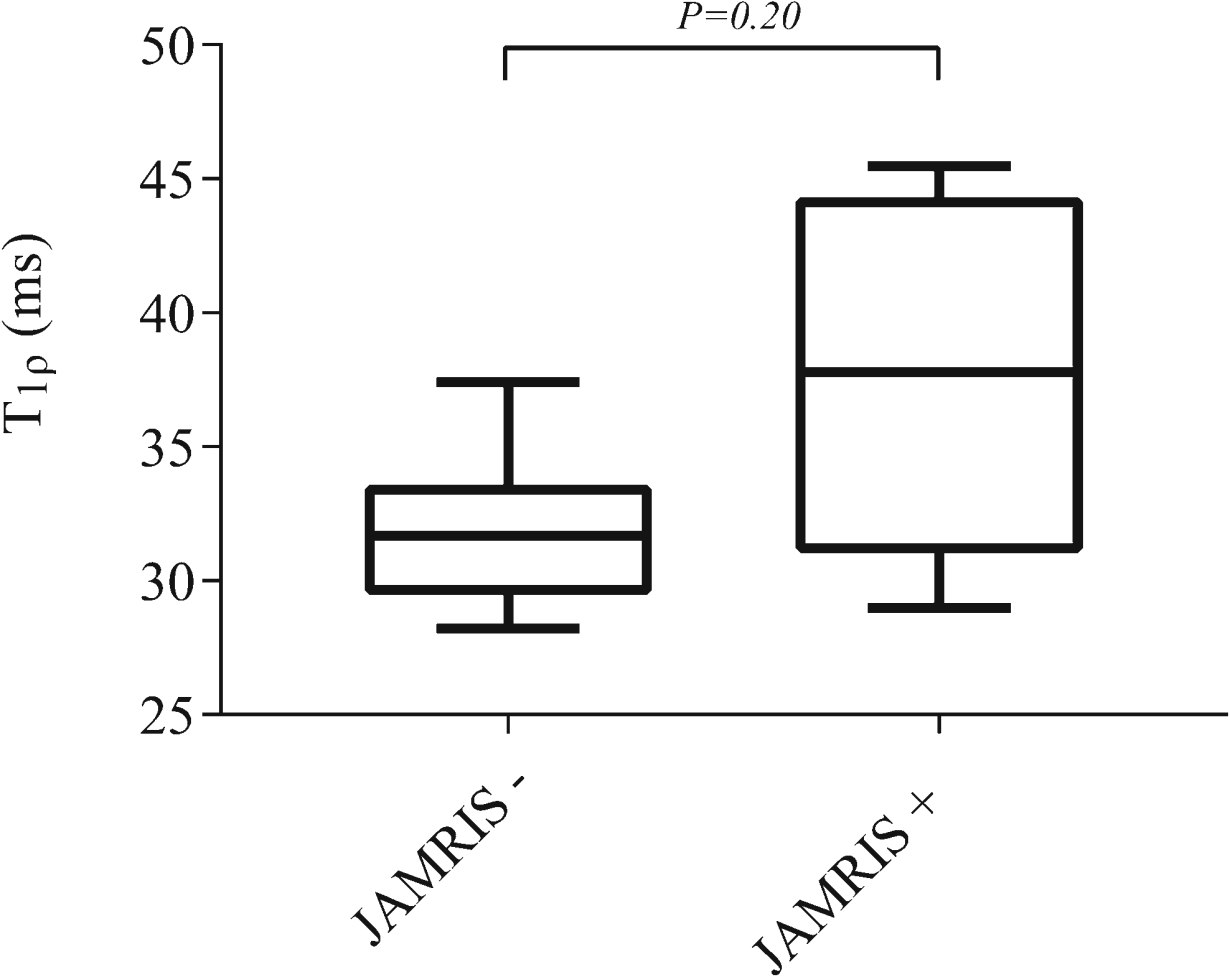
Boxplot shows the difference between T_1ρ_ values in MRI-inactive disease on the left and MRI-active disease on the right. *JAMRIS* juvenile arthritis MRI score. *P*<0.05 is significant

**Fig. 4:**
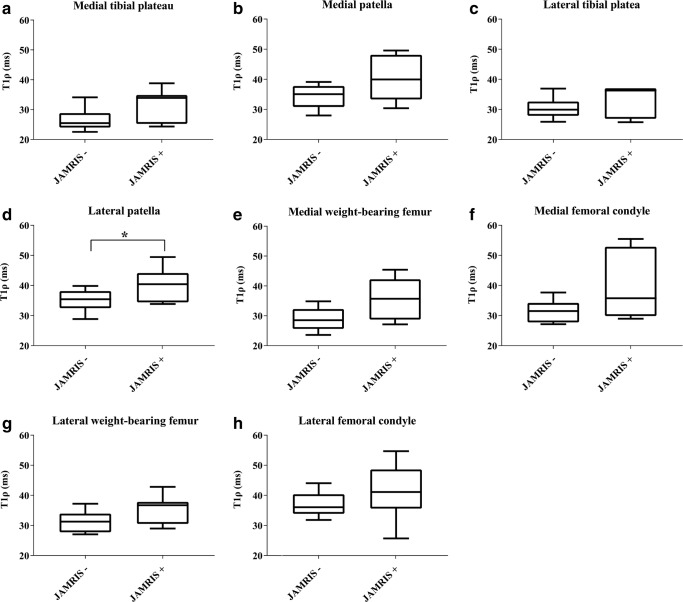
Boxplots of T_1ρ_ values by MRI-inactive disease (left in the boxplot, juvenile arthritis MRI score –) and MRI-active disease (right in the boxplot, juvenile arthritis MRI score +). **a**–**h** Readings within the medial tibial plateau (**a**), medial patella (**b**), lateral tibial plateau (**c**), lateral patella (**d**), medial weight-bearing femur (**e**), medial femoral condyle (**f**), lateral weight-bearing femur (**g**) and lateral femoral condyle (**h**). Consistently, all regions of interest show lower T_1ρ_ values for children with MRI-inactive disease. Significant difference was only seen in the lateral patella (*P*=0.046). *JAMRIS* juvenile arthritis MRI score

Morphologic cartilage damage (cartilage erosion or thinning of the cartilage) was not seen in any of the children on the conventional MR sequences. Children without inflammation on MRI (juvenile arthritis MRI score of 0, *n*=6) had a median T_1ρ_ value of 31.7 ms (IQR 29.5–33.6), while children with inflammation on MRI (juvenile arthritis MRI score of 1 or higher, *n*=7) had a median T_1ρ_ value of 37.8 ms (IQR 31.0–44.3; Fig. 3). This difference was not statistically significant (*P*=0.20). T_1ρ_ values of the eight individual regions of interest are presented in Table 3 and Fig. 4. T_1ρ_ values of the cartilage of the lateral patella were significantly lower in children without inflammation on MRI (35.4 ms, IQR 32.5–38.1) as opposed to children with inflammation on MRI (40.4 ms, IQR 34.4–44.1), *P*=0.046.

**Table 3:** Median (interquartile range) T_1ρ_ values of individual cartilage regions of interest in milliseconds

	T_1ρ_ in MRI-inactive disease (ms)	T_1ρ_ in MRI-active disease (ms)	*P* value
Weight-bearing			
Medial tibial plateau	25.5 (24.0–28.8)	33.9 (25.3–34.9)	0.199
Lateral tibial plateau	29.9 (27.9–32.6)	36.3 (26.9–37.0)	0.474
Medial weight-bearing femur	28.5 (25.6–32.2)	35.7 (28.8–42.2)	0.086
Lateral weight-bearing femur	31.3 (27.8–33.9)	36.7 (30.5–37.8)	0.116
Non-weight-bearing			
Medial patella	35.1 (30.8–37.7)	40.0 (33.3–48.1)	0.153
Lateral patella^a^	35.4 (32.5–38.1)	40.4 (34.4–44.1)	0.046
Medial femoral condyle	31.5 (27.7–34.2)	35.8 (29.8–52.9)	0.116
Lateral femoral condyle	36.0 (33.9–40.4)	41.1 (35.6–48.6)	0.253

A P-value <0.05 was considered to indicate a statistically significant difference

### Weight-bearing cartilage versus non-weight-bearing cartilage

Overall, weight-bearing cartilage had a significantly lower T_1ρ_ value compared to non-weight-bearing cartilage, with T_1ρ_ values for weight-bearing cartilage of 29.5 ms (IQR 27.6–36.0) and 34.8 ms (IQR 33.7–40.4) for non-weight-bearing cartilage, *P*-value=0.04.

### Correlation between T1ρ and disease activity parameters, age and body mass index

**Fig. 5:**
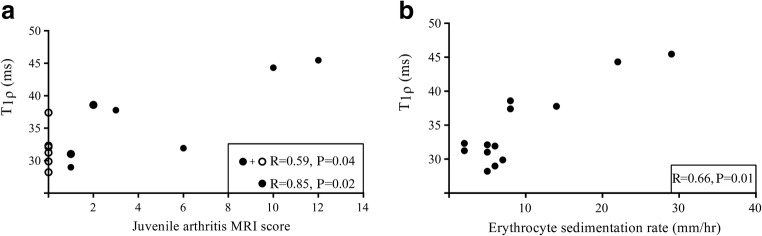
Plot shows correlation between T_1ρ_ and disease activity parameters. **a** Correlation between T_1ρ_ values and the juvenile arthritis MRI score. **b** Correlation between T_1ρ_ values and erythrocyte sedimentation rate. The correlation coefficient, *r*, and associated *P*-values are given in the plot. In (**a**) both the correlation for all children (*closed and open circles*) and the correlation for only those with juvenile arthritis MRI≥1 score (*closed circles*) are given. *P*<0.05 is significant

The Spearman correlation coefficient between T_1ρ_ and the juvenile arthritis MRI score was 0.59 (*P*=0.04). When the six children with a juvenile arthritis MRI score of 0 were excluded, correlation between T_1ρ_ and the juvenile arthritis MRI score was 0.85 (*P*=0.02; Fig. 5). T_1ρ_ and erythrocyte sedimentation rate had a correlation coefficient of 0.66 with a *P*-value of 0.01 (Fig. 5). No significant correlation was observed between T_1ρ_ and the number of actively inflamed joints (correlation coefficient 0.15, *P*-value 0.62). Also, patient age and body mass index percentile were not correlated with the T_1ρ_ value (correlation coefficient −0.5, *P*-value 0.09 and −0.07, *P*-value 0.81, respectively).

## Discussion

In this pilot study, we show the feasibility of T_1ρ_ for assessing knee cartilage integrity in children with juvenile idiopathic arthritis. All 13 children in the study underwent the T_1ρ_ acquisition protocol without discomfort and all images were of sufficient quality. None of the 13 children showed structural cartilage damage on conventional MRI. We found excellent repeatability for derivation of T_1ρ_ values using manual cartilage segmentations on the T_1ρ_ images.

Concerning the assessment of cartilage integrity, we found high correlation between T_1ρ_ values and the juvenile arthritis MRI score, an MRI-based disease activity score, in the seven children who had inflammation in the knee, but we also observed that T_1ρ_ values in children with actively inflamed knees were not different compared to T_1ρ_ values in those with non-inflamed knees. Nevertheless, cartilage of the lateral patellar region of interest demonstrated significantly higher T_1ρ_ values as compared to cartilage in children without knee inflammation on MRI. The patellar cartilage borders the patellofemoral synovium, which is often affected if knee arthritis is present [33]. Correspondingly, in our study, 4 of the 7 children with active arthritis indeed showed inflamed patellofemoral synovium. Thus, we hypothesize that increased T_1ρ_ values might represent pre-erosive microstructural damage to proteoglycans and collagen in the cartilage matrix that is not visualized using conventional MR sequences.

Several studies confirmed that T_1ρ_ values can be used to detect macrostructural and microstructural damage to cartilage in osteoarthritis [34–36]. In rheumatoid arthritis, the use of T_1ρ_ was first described in a study involving five people with rheumatoid arthritis [12]. In this study of cartilage specimens after total knee arthroplasty, T_1ρ_ values correlated with histological Safranin-O staining and macroscopic grade of severity of cartilage degeneration. In another study, radiocarpal cartilage was evaluated in a 3-month follow-up study of nine people with rheumatoid arthritis who used anti-rheumatic medication [37]. T_1ρ_ values correlated with treatment response, showing the potential of T_1ρ_ to measure changes in cartilage structure following treatment. Our results seem comparable with the findings in both osteoarthritis and rheumatoid arthritis [10, 12, 37] in which people with more severe disease activity were found to have higher T_1ρ_ values. When comparing the absolute T_1ρ_ values, we found (lowest-to-highest) 31 ms to 55 ms. Values in people with rheumatoid arthritis have been found to be 38–62 ms [12], and values in healthy pediatric patients 66–77 ms [17]. Note our values are somewhat lower. For the first comparison, this is probably attributable to the more severely affected cartilage in these children with rheumatoid arthritis who were scheduled for total knee arthroplasty. Another factor to take into account when comparing results from different studies is the spin lock frequency because T_1ρ_ values are higher at increased spin lock frequency. Our scans were acquired at lower frequency (400 Hz) than the scans of people with rheumatoid arthritis and healthy pediatric subjects (both acquired at 500 Hz) [12, 17].

We found a correlation between the juvenile arthritis MRI score and T_1ρ_ values as well as erythrocyte sedimentation rate and T_1ρ_ values. This supports the hypothesis that inflammation in the knee negatively affects the cartilage. We could not confirm our hypothesis that increasing age, and thus lower proteoglycan content, leads to lower T_1ρ_ values because we observed no correlation between age and T_1ρ_ values in this small cohort. However, this could be influenced by the age dispersion in our cohort because all but one child was older than 10 years.

Concerning body mass index, literature shows contradictory results. A recent study found correlation between body mass index and T_1ρ_ in the knee [38] while others decline a relation between body mass index and T_1ρ_ values in hip cartilage and intervertebral disc cartilage, respectively [39, 40]. In our study, body mass index was not correlated to T_1ρ_ values. It should be noted that our cohort consisted of mainly non-obese adolescents, thus we cannot exclude that age and body mass index could influence T_1ρ_ values in, for example, a 4-year-old or heavily obese child.

Limitations of this study are that none of the children had structural cartilage damage on MRI, hence it was not possible to examine the T_1ρ_ value in actual erosive cartilage damage. Moreover, histochemical proof of the hypothesized pre-erosive microstructural proteoglycan loss in the cartilage is lacking. However, obtaining cartilage specimens using biopsy is not feasible because this would harm the joints of these children. Furthermore, our T_1ρ_ experiments were performed at a spin lock frequency of 400 Hz because of specific absorption rate limitations and the need to keep the acquisition time short enough. Therefore, the used spin lock preparation did not completely remove all the contributions of dipolar interactions to the relaxation process. As a consequence, our readout is not completely specific to proteoglycan content and likely also reflects changes in the collagen matrix, such as degradation or swelling. Additional studies are needed in order to decouple the two contributions and gain more insight into the biochemical modifications induced by the disease. Another limitation is our segmentation. Although the cartilage segmentations were performed meticulously by an experienced reader, we cannot rule out that the cartilage–bone and cartilage–soft-tissue boundaries were imperfect. This could have influenced our results, especially if possible fluid pixels from joint effusion were wrongly included in the segmentation. To prevent this, we used three imaging planes when drawing the segmentations. Second, we performed our segmentation on full-thickness articular cartilage. Because cartilage has a zonal orientation, it would be interesting to subdivide the cartilage into a superficial and deep layer and study spatial variation in more detail. This could, for example, be performed using a normalization procedure to correct for different cartilage thicknesses in children, as has been done by authors studying cartilage with T2 relaxation time mapping in healthy children and children with juvenile idiopathic arthritis [41–44]. However, because our primary goal was to evaluate the feasibility of T_1ρ_, we did not perform such in-depth analyses of the cartilage; nevertheless, we would recommend a zonal analysis of the cartilage in studies that include a bigger sample of patients. Last, the small patient sample itself is considered a limitation and further work should focus on inclusion of more patients to validate the results of our pilot study. When more patients are included, the likelihood of scanning patients with structural cartilage damage would increase, which is important to affirm the assumption that higher T_1ρ_ values are seen in structurally abnormal cartilage as seen on conventional, qualitative MRI.

## Conclusion

There was acceptable feasibility and repeatability of T_1ρ_ for assessing knee cartilage in our sample. We found some association between T_1ρ_ values and erythrocyte sedimentation rate and the juvenile arthritis MRI score. In further studies, inclusion of more children — including children with erosive cartilage damage in the knee — is warranted to confirm the preliminary findings of this pilot study.
